# Association Between Parental Parenting Style Disparities and Mental Health: An Evidence From Chinese Medical College Students

**DOI:** 10.3389/fpubh.2022.841140

**Published:** 2022-02-28

**Authors:** Gan Ding, Lingzhong Xu, Long Sun

**Affiliations:** ^1^Centre for Health Management and Policy Research, School of Public Health, Cheeloo College of Medicine, Shandong University, Jinan, China; ^2^NHC Key Laboratory of Health Economics and Policy Research (Shandong University), Jinan, China

**Keywords:** parental parenting style, mental health, medical college students, cross-sectional study, parenting disparities

## Abstract

**Background:**

The associations between parental parenting styles and adolescents' development and health problems were also identified in a series of studies. However, the interactive impact of mother's and father's parenting style was less reported, which was implied in previous studies. In this study, we aim to analyze the associations between parental parenting style disparities and mental health among medical college students.

**Method:**

A cross-sectional study was conducted among medical college students in Shandong province, China, and 2,598 medical college students with parents were analyzed in this study. Items in a short form of Egna Minnen av Barndoms Uppfostran (EMBU) were used to calculate the parental parenting style disparities. Mental health was evaluated by the Kessler 10 scale.

**Results:**

The results of linear regressions showed that parental nurture reject disparities (RDs, β = 0.50, *p* < 0.001), parental emotional warmth disparities (WDs, β = 0.33, *p* < 0.001), parental overprotective disparities (ODs, β = 0.25, *p* < 0.001), and total disparities in parenting styles (TDs, β = 0.15, *p* < 0.001) were associated with mental health among medical college students, respectively. The other associated factors were age, ethnicity, chronic disease, above average family economic status, and good parental relationship.

**Conclusion:**

Our findings supported the positive association between parental parenting style disparities and mental health problems. Further studies can test the mechanism and intervention of the findings about the importance of parental parenting style consistence on mental health.

## Introduction

In the theories of attachment theory (1) and socialization theory (2, 3), parental parenting styles have been emphasized as a pivotal factor for children's and adolescents' social–emotional development. The associations between adolescents' development, health problems, and parental parenting styles were also identified in a series of studies (4–7). However, when we reviewed these studies, a majority of these reviews analyzed the roles of mother's and father's parenting styles separately (8–10).

We should recognize the importance of studies that explored the roles of parental parenting style. However, the interactive impact of mother's and father's parenting styles also should be paid attention (11). In this aspect, it should also be explored for the impact of the disparities between mother's and father's parenting styles on children and adolescents. As we know, in a family, mother and father were educated and developed in different families before they got married. The different education and development process for the mother and father may also result the different parenting styles when they parenting children (12). It further implies us that the parental style disparities between parents should be very common in the society (13). Considering the importance of parental style, we also have reason to believe that the kind of disparities may also have influences on their children' mental health.

Actually, there was some evidence, which had implied that the parental parenting style disparities might associate with children's and adolescents' mental health. First, in the theory of cognitive dissonance (14), cognitive dissonance is caused by two or more inconsistent notions or by the discrepancy between individuals' behaviors and values, which was an important risk factor for mental health (15, 16). Considering the importance of parental parenting style on children's and adolescents' cognitive development (17, 18), we have enough reasons to believe the impact of parental parenting style disparities on children's and adolescents' mental health. Second, many previous studies had identified the strong effect of parental characteristics on their children's mental health (19, 20). There were also some studies, which supported that mother played more important roles on children's mental health (21–23). These different roles of mothers and fathers also implied us that parental parenting style disparities may associate with children's mental health. Third, a study found that parental parenting style differences were associated with medical college students' suicidal ideation (24). Considering the strong associations between mental health and suicidal ideation (25, 26), we also have enough reasons to believe the association between parental parenting style differences and mental health. However, the association between parental parenting style disparities and mental health is less explored until now.

To fill the gap, we conducted a cross-sectional study in Chinese medical college students to analyze the association between parental parenting style disparities and mental health. As we know, medical college students were featured by the heavy study works, and they are also in higher risk of mental health problems (21). Considering the important role of mental health problems on the quality of the healthcare (27, 28), studies about mental health problems among medical college students are an important topic. It not only can help us to further understand the interactive impact of parental parenting style on mental health, but also can give suggestions to the parents on the progress of parenting.

## Methods

### Study Sample and Interviewing Procedures

This study was conducted among medical college students in Chinese Shandong province with a cross-sectional design. A multi-stage cluster sampling method was used to reach the medical college students. In Shandong province, there are 6 medical colleges, and we randomly selected 2 of them. In each selected medical college, all 12 medical majors were covered in this study. For each major, we selected one class from each grade to conduct the interview. Totally, we interviewed 2,723 medical college students in this study. However, considering the aims of this study, we deleted all the participants with single parent in the data analysis. Finally, we analyzed 2,598 medical college students in this study.

All of the interviewers were graduated students (major in public health) in Shandong University. Before the interview, all the interviewers were trained for 1 day to make sure that they had fully understood this study design and questionnaire. For the selected classes, the students were gathered in their classrooms. First, two interviewers introduced this study. After the students' agreement on the written informed consent, they were asked to fill the questionnaire by themselves. These two interviewers were in their classroom to answer the questions about this study and questionnaire, they also need to check each questionnaire to make sure the completeness of the questionnaires. In this study, all the students were not compensated with credits or awards in any way. The study protocol was approved by the Institutional Review Board (IRB) of Shandong University School of Public Health before data collection (ref. No.: 20181220).

### Measures

#### Mental Health

Mental health was evaluated by the Kessler-10 (K10) scale (29–31). This scale contains 10 items about anxiety and depressive symptoms in the past 4 weeks. These symptoms were tired out for no good reason, nervous, so nervous that nothing could calm you down, hopeless, restless or fidgety, so restless you could not sit still, sad or depressed, so depressed that nothing could cheer you up, everything was an effort, and worthless. The answers can be chosen from 1 (none of the time) to 5 (all of the time). Sum of the 10 items was analyzed in this study. The higher score represented the higher risk of mental health. The K10 scale is a good tool to evaluate mental health among different populations, and it was also widely used in many previous studies (32, 33). The Chinese version of K10 was also identified with good validity and reliability to evaluate mental health (34).

#### Parental Parenting Style Disparities

Parental parenting style disparities were calculated by a short form of Egna Minnen av Barndoms Uppfostran (EMBU) scale (35). This scale is widely used tool to evaluate parenting styles worldwide (36, 37). The Chinese version of this scale was also used in many previous studies (24, 38). In this study, the Cronbach's alpha for the EMBU scale was 0.855. In this version of EMBU, there were 21 items to evaluate the father's and mother's parenting styles, respectively. The 21 items were used to evaluate in three sub-scales (rejection, emotional warmth, and overprotection). There were 6 items in the rejection sub-scale (items 1, 4, 7, 12, 14, and 19), 7 items in the emotional warmth sub-scale (items 2, 6, 9, 11, 13, 17, and 21), and 8 items in the overprotection sub-scale (item 3, 5, 8, 10, 15, 16, 18, and 20). In this study, we calculated parental nurture reject disparity (RD), parental emotional warmth disparity (WD), parental overprotective disparity (OD), and total disparity in parenting styles (TD) by the following formulas, which was also used in previous study (24).


$$\begin{array}{l} \text{RD}=\underset{n=i}{\sum }|\alpha_{i}-\beta_{i}|\left(i=\text{item }1,\;4,\;7,\;12,\;14,\;19\right)\\ \text{WD}=\underset{n=j}{\sum }|\alpha_{j}-\beta_{j}|\left(j=\text{item }2,\;6,\;9,\;11,\;13,\;17,\;21\right)\\ \text{OD}=\underset{n=k}{\sum }|\alpha_{k}-\beta_{k}|\left(k=\text{item }3,\;5,\;8,\;10,\;15,\;16,\;18,\;20\right)\\ \text{TD}=\text{RD}+\text{WD}+\text{OD} \end{array}$$

where *i, j*, and *k* represent the item number in the EMBU. α_*i,j,k*_ and β_*i,j,k*_ represent father's and mother's scores in each item in the EMBU. After calculating the 21 items by these formulas, the higher scores of these disparities mean higher parental parenting style disparities, which were analyzed in this study.

#### Sociodemographic Variables

Gender was evaluated by male (1) or female (0). Age was calculated by the participants' data of birth. Ethnicity was assessed by the questions about the participants' ethnicity. As there were few ones, who are not Hans. We recoded in to Hans (1) and others (0). Chronic disease was assessed by a question that “Do you diagnose with any chronic diseases?” The answer could be chosen from “yes (1)” or “no (0).” Only child was evaluated by the question that “are you the only child in your family?”. The answer can be chosen from yes or no. Participants with positive answer were seen as only child (1) in their family. Family economic status was estimated by the question that “How about your family economic status, comparing with your classmates.” The answer can be chosen from very good, good, average, bad, and very bad. We recoded it into above average, average, and below average. The above average contained the first two answers (very good and good), and the below average contained the last two answers (bad and very bad).

#### Parental Characteristics

Parental education level was evaluated by father's and mother's academic level. The answers were illiteracy/semiliterate, elementary, junior school, technical secondary school, junior college, senior school, bachelor, and master and above. We recoded it into junior school or below (1), junior college/senior high school (2), and bachelor degree or above (3). Parental vocations were estimated by agricultural workers, workers, individual business, officer, teacher, and others. We recoded into agricultural workers and workers (1) and others (0).

### Statistical Methods

IBM SPSS Statistics 24.0 (Web Edition) was used for the data analysis. One-way ANOVA or *t*-tests were used to compare the mental health among or between different groups. The Pearson correlation was used to analyze the associations between continuous variables and mental health. Linear regression was performed to examine the associations between parental parenting style disparities and mental health, after controlling other variables. All of the tests were two-tailed, and a *p* < 0.05 was considered statistically significant.

## Result

Totally, the sample of 2,589 medical college students were analyzed in this study, and their sociodemographic and parental characteristics were listed in the first two columns of Table 1. Single analyses were also conducted to analyze the associations between sociodemographic characteristic, parental characteristic, and mental health. The results supported that age (*r* = 0.10, *p* < 0.001), ethnicity (*t* = −2.17, *p* < 0.05), chronic disease (*t* = 6.11, *p* < 0.001), family economic status (*F* = 11.69, *p* < 0.001), and parental relationship (*F* = 18.31, *p* < 0.001) were associated with mental health. The detailed information is shown in Table 1.

**Table 1:** Description and single analyses for the association between sociodemographic factors and mental health.

**Variables**	**Total, *N* (%)**	**Mental health (mean ±SD)**	***F/t/r***
Observations	2,598 (100.0)	19.15 ± 6.64	
Gender			*t* = 1.00
Male	1,162 (44.7)	19.30 ± 6.97	
Female	1,436 (55.3)	19.04 ± 6.37	
Age (mean ± SD)	20.42 ± 1.27	–	*r* = 0.10^***^
Ethnicity			*t* = −2.17^*^
Hans	2,518 (96.9)	19.10 ± 6.60	
Others	80 (3.1)	20.74 ± 7.65	
Chronic disease			*t* = 6.11^***^
Yes	116 (4.5)	22.81 ± 7.00	
No	2,482 (95.5)	18.98 ± 6.58	
Family economic status			*F* = 11.69^***^
Above average	524 (20.2)	18.22 ± 6.81	
Average	1,795 (69.1)	19.21 ± 6.53	
Below average	279 (10.7)	20.57 ± 6.76	
Only child			*t* = −1.26
Yes	1,197 (46.1)	18.98 ± 6.87	
No	1,401 (53.9)	19.31 ± 6.44	
Parental relationship			*F* = 18.31^***^
Good	2,183 (84.0)	18.82 ± 6.50	
Normal	337 (13.0)	20.81 ± 6.83	
Bad	78 (3.0)	21.47 ± 8.05	
Father's education level			*F* = 2.39
Junior high school or below	1,320 (50.8)	19.43 ± 6.32	
Junior college/senior high school	893 (34.4)	18.85 ± 6.84	
Bachelor degree or above	385 (14.8)	18.90 ± 7.21	
Mother's education level			*F* = 2.14
Junior high school or below	1,573 (60.5)	19.35 ± 6.51	
Junior college/senior high school	723 (27.8)	18.99 ± 6.69	
Bachelor degree or above	302 (11.6)	18.55 ± 7.15	
Father's vocation			*t* = 1.40
Agricultural workers and workers	1,280 (49.3)	19.34 ± 6.35	
Others	1,318 (50.7)	18.98 ± 6.91	
Mother's vocation			*t* = 1.74
Agricultural workers and workers	1,232 (47.4)	19.39 ± 6.49	
Others	1,366 (52.6)	18.94 ± 6.77	

* *p < 0.05*;

*** *p < 0.001*.

We further conducted bivariate correlation to analyze the association between parental parenting style disparities and mental health. For parental parenting style disparities, we calculated the RDs, WDs, ODs, and TDs based on the scores of EMBU. We found that RDs (*r* = 0.16, *p* < 0.001), WDs (*r* = 0.13, *p* < 0.001), ODs (*r* = 0.11, *p* < 0.001), and TDs (*r* = 0.15, *p* < 0.001) were associated with mental health. The correlation coefficient matrix of parental parenting style disparities and mental health is listed in Table 2.

**Table 2:** Correlation coefficient matrix for parental parenting style disparities and mental health.

**Variables**	**Mean ±SD**	**MH**	**RD**	**WD**	**OD**
MH	19.15 ± 6.64	–	–	–	–
RD	1.14 ± 1.90	0.16^***^	–	–	–
WD	1.44 ± 2.25	0.13^***^	0.64^***^	–	–
OD	1.76 ± 2.61	0.11^***^	0.68^***^	0.68^***^	–
TD	4.34 ± 5.97	0.15^***^	0.86^***^	0.88^***^	0.91^***^

*MH denotes mental health, RD denotes parental nurture reject disparity, WD denotes parental emotional warmth disparity, OD denotes parental overprotective disparity, and TD denotes total disparity in parenting styles*.

*** *p < 0.001*.

In Table 3, multiple linear regressions are further performed to identify the factors associated with mental health in 4 models. In Model A, mental health was associated with age (β = 0.46, *p* < 0.001), Hans ethnicity (β = −1.52, *p* < 0.05), chronic disease (β = 3.43, *p* < 0.001), above average family economic status (β = −0.98, *p* < 0.01), good parental relationship (β = −1.35, *p* < 0.001), and RDs (β = 0.50, *p* < 0.001). In Model B, mental health was associated with age (β = 0.47, *p* < 0.001), Hans ethnicity (β = −1.61, *p* < 0.05), chronic disease (β = 3.41, *p* < 0.001), above average family economic status (β = −0.93, *p* < 0.01), good parental relationship (β = −1.46, *p* < 0.001), and WDs (β = 0.33, *p* < 0.001). In Model C, mental health was associated with age (β = 0.49, *p* < 0.001), Hans ethnicity (β = −1.63, *p* < 0.05), chronic disease (β = 3.44, *p* < 0.001), above average family economic status (β = −0.89, *p* < 0.01), good parental relationship (β = −1.55, *p* < 0.001), and ODs (β = 0.25, *p* < 0.001). In Model D, mental health was associated with age (β = 0.48, *p* < 0.001), Hans ethnicity (β = −1.55, *p* < 0.05), chronic disease (β = 3.39, *p* < 0.001), above average family economic status (β = −0.96, *p* < 0.01), good parental relationship (β = −1.39, *p* < 0.001), and TDs (β = 0.15, *p* < 0.001).

**Table 3:** Linear regression for the association between parental parenting style disparities and mental health (β and its 95% CI).

**Variables**	**Model A**	**Model B**	**Model C**	**Model D**
Male	0.25 (−0.27, 0.78)	0.40 (−0.12, 0.93)	0.37 (−0.16, 0.89)	0.32 (−0.21, 0.84)
Age	0.46 (0.27, 0.66)^***^	0.47 (0.27, 0.67)^***^	0.49 (0.29, 0.69)^***^	0.48 (0.28, 0.68)^***^
Hans ethnicity	−1.52 (−2.97, −0.08)^*^	−1.61 (−3.06, −0.16)^*^	−1.63 (−3.09, −0.18)^*^	−1.55 (−3.00, −0.10)^*^
Chronic disease	3.43 (2.22, 4.64)^***^	3.41 (2.19, 4.62)^***^	3.44 (2.23, 4.66)^***^	3.39 (2.18, 4.60)^***^
Family economic status (Ref. = Average)				
Above average	−0.98 (−1.64, −0.31)^**^	−0.93 (−1.60, −0.26)^**^	−0.89 (−1.56, −0.22)^**^	−0.96 (−1.62, −0.29)^**^
Below average	0.44 (−0.41, 1.30)	0.43 (−0.43, 1.28)	0.44 (−0.42, 1.30)	0.42 (−0.43, 1.27)
Only child	−0.41 (−0.97, 0.15)	−0.46 (−1.02, 0.11)	−0.43 (−0.99, 0.14)	−0.46 (−1.03, 0.10)
Parental relationship (Ref. = Normal)				
Good	−1.35 (−2.11, −0.58)^***^	−1.46 (−2.23, −0.69)^***^	−1.55 (−2.31, −0.79)^***^	−1.39 (−2.15, −0.62)^***^
Bad	0.35 (−1.26, 1.95)	0.17 (−1.44, 1.79)	0.30 (−1.31, 1.92)	0.27 (−1.33, 1.88)
Father's education level (Ref = Junior high school or below)				
Junior college/Senior high school	−0.34 (−0.97, 0.30)	−0.25 (−0.89, 0.39)	−0.28 (−0.92, 0.36)	−0.28 (−0.92, 0.36)
Bachelor degree or above	0.24 (−0.82, 1.29)	0.21 (−0.85, 1.26)	0.20 (−0.86, 1.26)	0.22 (−0.84, 1.28)
Mother's education level (Ref = Junior high school or below)				
Junior college/Senior high school	0.15 (−0.54, 0.85)	0.15 (−0.55, 0.85)	0.14 (−0.55, 0.84)	0.15 (−0.55, 0.84)
Bachelor degree or above	−0.48 (−1.62, 0.66)	−0.29 (−1.43, 0.85)	−0.34 (−1.48, 0.81)	−0.37 (−1.50, 0.77)
Father's vocation (Agricultural workers and workers)	−0.32 (−1.06, 0.43)	−0.31 (−1.06, 0.44)	−0.31 (−1.06, 0.44)	−0.32 (−1.06, 0.43)
Mother's vocation (Agricultural workers and workers)	0.21 (−0.53, 0.94)	0.24 (−0.50, 0.97)	0.26 (−0.48, 0.99)	0.24 (−0.50, 0.97)
RD	0.50 (0.37, 0.64)^***^	–	–	–
WD	–	0.33 (0.21, 0.44)^***^	–	–
OD	–	–	0.25 (0.16, 0.35)^***^	–
TD	–	–	–	0.15 (0.10, 0.19)^***^
Constant	12.01 (7.70, 16.33)^***^	12.05 (7.72, 16.39)^***^	11.82 (7.47, 16.17)^***^	11.71 (7.39, 16.04)^***^
*R* ^2^	0.06	0.06	0.05	0.06

*RD denotes parental nurture reject disparity, WD denotes parental emotional warmth disparity, OD denotes parental overprotective disparity, and TD denotes total disparity in parenting styles*.

* *p < 0.05*;

** *p < 0.01*;

*** *p < 0.001*.

## Discussion

In this study, we analyzed the association between parental parenting style disparities and mental health among Chinese medical students, and we found that all kinds of the parental parenting style disparities (RD, WD, OD, and TD) was positively associated with mental health problems. The other factors associated with medical college students' mental health were older age, Hans ethnicity, chronic disease, not above average family economic status, and no good parental relationship.

The main aim for this study was to identify the association between parental parenting style disparities and mental health, and the association between them was supported in this study. To our knowledge, this is the first study to explore the association between parental parenting style disparities and mental health. It is also helpful for us to further understand the effect of parental parenting style on mental health. As we know, cognitive dissonance was an important risk factor for mental health, and the different parenting styles between mother and father may cause cognitive dissonance to their children (39, 40). This may be one of the reasons to explain the findings. In the other side, previous studies also found that authoritative parenting style was negatively associated with mood problems (41). As we know, authoritative parenting style was characterized by that both parents' reflectiveness in terms of their children's psychosocial needs and adequate parental monitoring (42). It also means that both mother and father are demanding and responsive to their children. It further implied us the importance of parenting consistence between mothers and fathers on children' mental health.

In this study, we also found that all the RD, WD, OD, and TD were positively associated with mental health problems. As we know, previous studies had identified that the nurture reject or overprotection parenting style was positively associated with mental health problems (43), whereas the emotional warmth parenting style was negatively associated with mental health problems (44). The different associations implied us that the parental parenting disparities were independently associated with mental health regardless of the risk or protective effect of parenting styles on mental health. It further implied us that the parents should bring into correspondence with each other for their parenting styles.

We also found that older age, other ethnicity, chronic disease, no above average family economic status, and no good parental relationship were positively associated with mental health problems among medical college students. Actually, all these factors had been identified in previous studies (45–47). For age, it may be caused by the higher academic stress for medical college students in higher grades (48). For ethnicity, it may be caused by lack of peer support for other ethnicity students (49, 50) because of the small percentage of them. Comparing with an average family economic status, students with an above average economic status were in better mental health. It may be explained by the influence of social economic status, which was supported in previous studies (51). Students with good parental relationship were in better mental health, comparing with normal parental relationship. It was also easily understood and supported in previous studies.

There were some limitations, which should be considered when we interpret the findings in this study. First, this is a cross-sectional study, and we cannot find any causal relationship between parental parenting style disparities and mental health. Second, although parental parenting style disparities were calculated by a standardized scale, a scale about the parental parenting style disparities can be developed and used to evaluate parental parenting style disparities. Third, the data analyzed in this study was collected by self-reported, and some variables were evaluated by one question. Both of these may bring some bias to the results. Finally, the findings in this study was based on the medical college students in two medical colleges, a more comprehensive sample can be analyzed to get a more credible result.

Keeping these limitations in mind, we can also conclude the associations between parental parenting style disparities and mental health. The results also recommend that we cannot only consider the effect of parental parenting styles on mental health, but the parenting style disparities between mother and father should also be paid attention. The results also support the interactive and incorporating associations for the mother's and father's parenting style. Further studies can test the mechanism and intervention of the findings about the importance of parental parenting style consistence on mental health.

## Data Availability Statement

The raw data supporting the conclusions of this article will be made available by the authors, without undue reservation.

## Ethics Statement

The studies involving human participants were reviewed and approved by Institutional Review Board (IRB) of Shandong University School of Public Health. The patients/participants provided their written informed consent to participate in this study.

## Author Contributions

GD analyzed the data and wrote the draft. LX commented on the manuscript. LS designed this study and revised the manuscript. All authors read and approved the final manuscript.

## Funding

The research was supported by the National Natural Science Foundation of China (71603149 and 71974114).

## Conflict of Interest

The authors declare that the research was conducted in the absence of any commercial or financial relationships that could be construed as a potential conflict of interest.

## Publisher's Note

All claims expressed in this article are solely those of the authors and do not necessarily represent those of their affiliated organizations, or those of the publisher, the editors and the reviewers. Any product that may be evaluated in this article, or claim that may be made by its manufacturer, is not guaranteed or endorsed by the publisher.
